# Three-Month Retention of Basic Life Support with an Automated External Defibrillator Using a Two-Stage versus Four-Stage Teaching Technique

**DOI:** 10.1155/2019/1394972

**Published:** 2019-07-16

**Authors:** Katrine Bjørnshave Bomholt, Lise Qvirin Krogh, Svend Rosendahl Bomholt, Mette Amalie Nebsbjerg, Troels Thim, Bo Løfgren

**Affiliations:** ^1^Emergency Department, Aarhus University Hospital, Palle Juul-Jensens Boulevard 161, 8200 Aarhus N, Denmark; ^2^Research Center for Emergency Medicine, Aarhus University Hospital, Palle Juul-Jensens Boulevard 161, 8200 Aarhus N, Denmark; ^3^Department of Obstetrics and Gynaecology, Regional Hospital of Herning, Gl. Landevej 61, 7400 Herning, Denmark; ^4^Department of Respiratory Diseases and Allergy, Aarhus University Hospital, Palle Juul-Jensens Boulevard 99, 8200 Aarhus N, Denmark; ^5^Department of Internal Medicine, Regional Hospital of Horsens, Sundvej 30, 8700 Horsens, Denmark; ^6^Department of Cardiology, Aarhus University Hospital, Skejby, Palle Juul-Jensens Boulevard 99, 8200 Aarhus N, Denmark; ^7^Institute of Clinical Medicine, Aarhus University Hospital, Skejby, Palle Juul-Jensens Boulevard 99, 8200 Aarhus N, Denmark; ^8^Department of Internal Medicine, Regional Hospital of Randers, Skovlyvej 1, 8930 Randers, Denmark

## Abstract

**Introduction:**

Resuscitation training increases bystander's ability to perform basic life support (BLS) with an automated external defibrillator (AED) immediately after training. However, several studies indicate that resuscitation skills decay rapidly.

**Methods:**

This study evaluates retention of BLS/AED skills three months after an initial study comparing acquisition of BLS/AED skills among laypersons immediately after training with a two-stage versus four-stage teaching technique.

**Results:**

There was no difference in retention of BLS/AED skills (pass rate 10.8% versus 10.9%, respectively, p=1) three months after training. Total average number of skills adequately performed (of 17) was 13.3 versus 13.7 among laypersons trained with a two-stage and a four-stage technique, respectively. No difference was found in quality of chest compressions and rescue breaths between the two groups.

**Conclusion:**

Three months after training, this study found no difference in retention of BLS/AED skills among laypersons taught using a two-stage compared to a four-stage teaching technique.

## 1. Introduction

Resuscitation training increases bystander's ability to adequately perform basic life support (BLS) and to use an automated external defibrillator (AED) immediately after initial training [1, 2]. However, several studies indicate that resuscitation skills decay rapidly after initial training [1, 3]. Hence, qualified suggestions for improvement in retention of resuscitation skills are warranted.

Different teaching techniques for practical skills are used. A four-stage teaching technique is a widely accepted teaching technique integrated in educational programs. It breaks down the skill teaching process into four phases: demonstration, deconstruction, formulation, and performance. The traditional class room training requires a great deal of instructor time and expense. Reduction of this four-stage technique to a two-stage (“see one, do one”) technique can shorten course duration and thereby result in cost effective and time efficient resuscitation training [4].

In previous studies, the four-stage teaching technique has not been proven to be superior to a two-stage teaching technique when teaching relatively simple skills [4]. We compared acquisition of BLS/AED skills and self-confidence in BLS/AED skills with high complexity among laypersons immediately after instructor-led training with a two-stage versus four-stage teaching technique and found that two-stage teaching technique was noninferior to the four-stage teaching technique immediately after training [5].

This study reports the retention of BLS/AED skills three months after teaching laypersons BLS/AED using a two-stage versus a four-stage teaching technique.

## 2. Methods

### 2.1. Study Design

This study evaluates retention of BLS/AED skills after a prospective, controlled randomised noninferiority study. The initial study compared acquisition of BLS/AED skills and self-confidence in BLS/AED skills among laypersons immediately after instructor-led training with a two-stage versus four-stage teaching technique. Participants were tested three months after course completion, to assess retention and self-confidence in BLS/AED skills in the two groups.

### 2.2. Participants and Training

From December 2012 to March 2013, nonhealthcare volunteers were enrolled in the initial study. Participants were randomised 1:1 to instructor-led course in single rescuer resuscitation of adults according to the ERC Guidelines for Resuscitation 2010 using either the two-stage or the four-stage teaching technique. In brief, Stage 1: instructors demonstrate how the skill is performed at its original speed without commentary. Stage 2: instructors repeat the skill, describing and explaining all the theory behind facts and details. Stage 3: students guide the instructor through the skills while the instructor performs according to the student's instructions. Stage 4: students demonstrate and comment the skill procedure. Participants, randomised to the two-stage technique, were trained using a two-stage approach consisting of Stage 2 and Stage 4 as described above. Data for retention and self-confidence in BLS/AED skills of this study was collected during March to June 2013 testing the same participants.

### 2.3. Test Scenario

Participants were tested after three months in a simulated cardiac arrest scenario with the same setup as the test immediately after course completion to evaluate the retention of BLS/AED skills. Each test was recorded on video. The manikin was connected to a laptop computer sampling data of chest compressions and rescue breaths. After the assessment, participants completed a questionnaire on self-confidence as BLS/AED providers on a five-point Likert scale.

### 2.4. Skills Assessment and Test Measurements

Skills were evaluated by two blinded assessors based solely on review of video recordings of the tests. Skills were evaluated in accordance with the ERC Guidelines for Resuscitation 2010 covering 17 actions representing individual steps of the BLS/AED algorithm as adequately or inadequately performed. Passing required that 17 of the 17 actions were rated as adequately performed.

The quality of chest compressions and rescue breaths were analysed from video recordings and from data collected from the resuscitation manikin as described previously [5].

### 2.5. Statistics

The sample size of the initial study was 80 participants in each group based on a sample size calculation pertaining to the primary endpoint in that study [5].

For continuous variables, we used student t-test or Mann Whitney test according to data distribution. For the remaining variables, we used Chi2-test or Fishers exact test. To compute 95% confidence intervals on differences between proportions, we used the standard approximations using the Chi2-test with Yates correction and these intervals should be interpreted with caution. Calculations were conducted using GraphPad Prism (Version 6.00 for Windows, GraphPad, La Jolla, CA, USA). A P-value <0.05 was considered statistically significant.

## 3. Results

**Figure 1:**
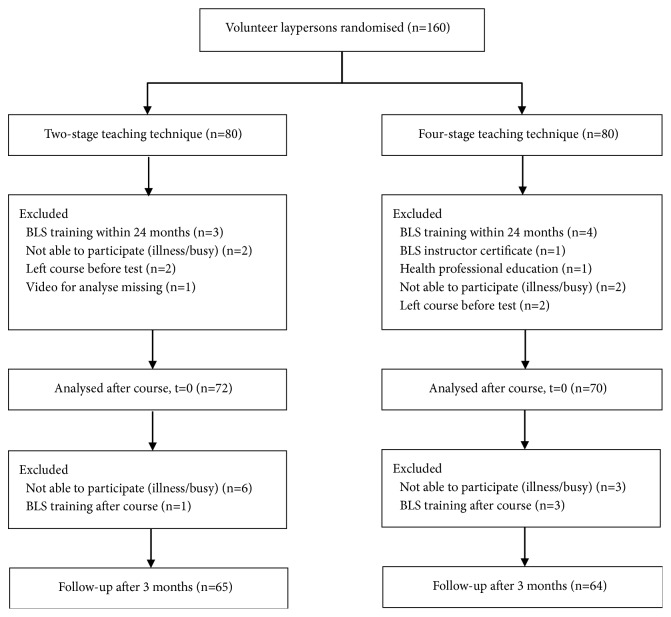
Participant flow diagram.

A total of 160 participants were included in the study (Figure 1). The number of participants lost to training or evaluation immediately after course completion was 8 versus 10, and additionally 7 versus 6 were lost to follow-up in the two-stage and four-stage teaching groups, respectively. There were no differences in baseline demographics of the participants (Table 1). The interval between training and test of retention were 2.9 (1.7) versus 2.5 (1.7) days from three months in the two groups, respectively.

No difference was found in pass rate immediately after course completion (pass rate 56.9% versus 58.6%, respectively, p=0.87) [5]. There was no statistical significant difference in retention of BLS/AED skills three months after training with a pass rate of 10.8% versus 10.9%, respectively, p=1.

Total average skills adequately performed (of 17) decayed from 16.1 versus 16.2 to 13.3 versus 13.7 among laypersons trained with the two-stage (n=65) and the four-stage (n=64) technique, respectively. [5] There were no significant differences between the two groups in any of the individual skills except ensuring the safety (p=0.015) (Table 2). Skills that were most difficult to perform adequately were initial airway opening, initial airway check, chest compressions, correct sequence, and safe AED analysis.

No differences were found in quality of chest compressions and rescue breaths between the two groups (Table 3). Due to technical difficulties with the manikin and electronic sampling, some data are missing for tidal volume (n = 8 and n = 6 for two- and four-stage, respectively).

Most study participants felt capable of performing BLS/AED three months after course completion (98% versus 97% in two- versus four-stage teaching technique)

## 4. Discussion

The main finding of this study was that there was no difference in retention of BLS/AED skills three months after training among laypersons taught using a two-stage teaching technique compared to a four-stage teaching technique. This study supports the results of previous studies with similar learning outcome with the four-stage and two-stage teaching techniques [4, 5]. There were no differences in both acquisition and retention of more complex skills including both BLS and AED. A two-stage teaching technique may be a time-effective alternative when teaching both health care professionals and laypersons both relatively simple and more complex practical skills.

There was a marked decrease in skill level over the first three months after training. Skills that were most difficult to perform adequately were initial airway opening and check for breathing, chest compressions, correct sequence, and safe AED analysis. In this study, pass rates decreased over the first three months with 46 % (from 57% to 11%) after two-stage and 48% (from 59% to 11%) after four-stage teaching technique training. The low pass rates are related to the deterioration in retention of the skills [5]. However, this result should also be considered in the light of the very strict threshold of passing the test (17 correctly performed skills out of 17 possible). A three-month interval for follow-up was selected because previous studies have shown that CPR skills can decay significantly in as little as 3-6 months [2, 3]. To maintain adequate resuscitation skills, refresher training in BLS/AED is required. However, the optimal timing for refresher training is not yet determined. Currently the recommendations on first time refresher training are variable, going from 6 to 24 months after initial training [6, 7]. The optimal type of refresher training to maintain adequate resuscitation skills is not determined so far [6, 7]. Refresher training should address skill retention, and in order to be effective, refresher training should be easily accessible and available at with no or low cost, making it more likely to be used by the general population. E-learning meets these requirements and is an increasingly popular teaching modality.

Bystanders often refrain from performing BLS in real life due to lack of self-confidence in BLS skills. BLS training increases self-confidence and willingness to perform bystander CPR [8]. Improvements in self-confidence and comfort level in providing BLS/AED is therefore of importance when selecting training method. Most study participants agreed that they felt capable of performing BLS/AED three months after course completion irrespective of the training technique.

## 5. Conclusion 

Three months after BLS/AED training, this study found no difference in retention of BLS/AED skills among laypersons taught using a two-stage teaching technique compared to a four-stage teaching technique. Future studies should investigate and help develop methods to improve the performance among those who fail to achieve an appropriate skill level after training, regardless of training technique.

## Figures and Tables

**Table 1:** Baseline demographics.

	Two-stage	Four-stage
	(n=65)	(n=64)
Age (years)	42 (13)	40 (10)

Sex		
Female	43 (66%)	45 (70%)

Level of education		
Primary and lower secondary school	2 (3%)	1 (2%)
Gymnasium (preuniversity)	2 (3%)	3 (5%)
Craftsman	3 (5%)	8 (13%)
Higher education (≤2 years)	15 (23%)	12 (19%)
Higher education (2-4.5 years)	27 (42%)	26 (41%)
Higher education (≥4.5 years)	16 (25%)	14 (22%)

Age is reported as mean (SD) and remaining variables as n (%).

Total percentage of level of education in two stages is 101 % and in four stages 102 % due to rounding

**Table 2:** BLS/AED skills performed adequately.

	Two-stage	Four-stage	P-value
	(n=65)	(n=64)	
*Skills*			

*BLS*			
Ensure safety	60 (92%)	48 (75%)	0.015
Checks for responsiveness	56 (86%)	59 (92%)	0.41
Shout for help	62 (95%)	63 (98%)	0.62
Initial airway opening	40 (62%)	42 (66%)	0.76
Initial breathing check	34 (52%)	40 (63%)	0.32
Call for help	61 (94%)	61 (95%)	0.98
Adequately chest compression	38 (57%)	43 (67%)	0.40
Adequately rescue breaths	49 (75%)	48 (75%)	0.88
Perform CPR (30:2) without interruptions	63 (97%)	57 (89%)	0.16
Sequence in order	29 (45%)	29 (45%)	0.92

*AED*			
Check if cardiac arrest	32 (49%)	39 (61%)	0.25
Switch on AED	65 (100%)	64 (100%)	-
Attach pads correctly	57 (88%)	60 (94%)	0.38
Safe AED analysis	42 (55%)	39 (61%)	0.80
Safety shock	55 (85%)	56 (88%)	0.83
Follow AED instructions	65 (100%)	64 (100%)	-
Perform CPR (30:2) without interruptions	59 (91%)	62 (97%)	0.28

Individual skills are presented as n(%).

**Table 3:** Quality of chest compressions and ventilation.

	Two-stage	Four-stage	P-value
	(n=65)	(n=64)	
*Chest compressions *			
Chest compressions rate (min^−1^)	108 (19)	107 (17)	0.78
Chest compressions number	30 (3.1)	29 (2.8)	0.21
Chest compression with correct hand placement (n (%))	54 (83%)	56 (88%)	0.62
Average compression depth (mm)	43 (11)	46 (10)	0.12
No-flow time (sec)	27 (11)	32 (19)	0.07

*Rescue breaths*			
Numbers of rescue breaths	2.0 (0.5)	2.2 (0.4)	0.10
Number of sufficient rescue breaths	1.6 (0.8)	1.6 (0.7)	0.62
Tidal volume (L)*∗*	0.7 (0.4)	0.6 (0.4)	0.16

Individual skills are presented as n (%) and mean (SD). *∗*Tidal volume n = 57 versus 58 in the two groups

## Data Availability

The data used to support the findings of this study are available from the corresponding author upon request.
